# Scientific Items

**Published:** 1886-12-20

**Authors:** 


### SCIENTIFIC ITEMS.

**A Dog's Reasoning.**— A recent English writer gives the following interesting example of a dog's reasoning from false premises:

"A Highland shepherd possessed a collie which he had reared from puppydom. He was exceedingly kind to the animal, and it is needless to say that the dog loved him, and was his constant companion day and night. In the Highland hills and glens — up Loch Carron way, where the event I am about to relate took place — snow storms are frequent in winter. It is the shepherd's duty then to look well after his flock, lest any get buried, and, if necessary, to drive them down to the lower land.

"It was with the latter intention that D—— took the hills one day with his dog. It was calm in the morning, but a wild snow storm was raging in the mountain before sunset. It will never be known whether or not the shepherd lost himself; the probability is that he sat down to rest in the lee of a rock, and fell into a deep sleep, from which he awoke no more.

"Next day the dog appeared at the man's cot; he was very excited, and did what he never did before — snatched a bannock from the table, and rushed out with it. The opportunity to follow the dog was not neglected, for collies will often bring assistance to a wounded master who has fallen over a rock or cliff.

"They tracked the poor collie through the snow, up the glen, and over the hills, till at last they found him sitting wofully by the side of his dead master. But the bannock? It was found laid up against the shepherd's cheek, the dog evidently having imagined it was from hunger his master suffered. Many a time and oft had that Highland shepherd shared his frugal meal with his collie — in autumn, when the bloom was on the hills; in winter and in spring, when sleet or rain or snow was borne along on the wings of the fierce, cold winds — and 'Now,' the collie must have thought, 'I must try to do something for my poor master, who lies so chill and still.'"— *Popular Science News.*

**Parasites of the House Fly.**—In an article on the supposed parasites seen with a microscope on the fly (*Pop. S. News*) we learn that the supposed parasites of the fly are not really parasites at all, as they do not draw any nourishment from his body, but are only travelers, or insect tramps, who have boarded the first available means of conveyance, and are "beating" their way to a locality where food and the conditions of existence are more favorable. These facts readily explain how fresh cheese can become infected with mites, even when carefully placed upon a shelf away from all supposed sources of contamination. A casual fly, alighting upon it, may detach from his body a number of the little insects, sufficient to convert the entire cheese into a scene of most undesirable activity in a very short time. In view of this additional misdemeanor of which the house fly has been proved guilty, it is not much satisfaction to know that the supposed parasites are not the cause of any harm, or even inconvenience, to the annoying insect upon whom they take passage.

**The** element fluorine, which has heretofore defied all efforts to isolate it from its compounds, has at last been produced by a French chemist, M. Moissan. It was obtained by decomposing, by an electric current from twenty Bunsen cells, a quantity of anhydrous fluoric acid contained in a plattinum U-tube, at a temperature of —23° C. Hydrogen gas was given off at the negative pole, and was readily recognized; while the other arm of the tube became filled with a gas which was undoubtedly pure fluorine, as the results of an analysis show that it contains no hydrogen. As might have been anticipated, fluorine is extremely active, violently attacking organic substances, and uniting with various elements to form fluorides. Water is decomposed by it into ozone and hydrofluoric acid. The correspondent of the *Druggists' Circular*, to which journal we are indebted for this information, says that M. Moissan was at work upon the problem for nearly a year before his efforts were rewarded by this brilliant discovery.—*Ibid.*

**Some** years ago a certain Dr. Tanner obtained considerable notoriety by undergoing a fast of forty days, during which time he apparently took nothing into his stomach but pure water. A similar fast of thirty days has just been completed in Italy by a Signor Succi, who claims to have discovered a plant growing in Africa by the aid of which a man can sustain himself without food for weeks, or even months, at a time Although this fast is probably only an advertisement of some patent "nerve food," it seems to have been honestly conducted. Succi took exercise constantly during its continuance, and at the end was in much better condition than might have been expected. It is hardly necessary to remark that such exceptional cases are not to be considered as indicating the capacity of the average man to endure starvation, but simply as feats of remarkable endurance, of little or no scientific value.—*Popular Science News.*

**At** the Cape of Good Hope, near Table Mountain, the clouds come down very low and then disappear without dropping in rain. At sueh a time, if a traveler should go under a tree for shelter from the threatening storm, he would find himself in a drenching shower, while out in the open space, away from any tree or shrub, everything would be as dry as a bone. The cloud or mist is rather warmer than the leaves, and so, when it touches them, it changes into clinging drops, which look like dew. Fresh drops keep forming; they run together; and, at length, the water drips off the leaves like rain. And this process goes on until the clouds lift and the sun comes out again.—*Ex.*

**Nickel Poison.**—An order has been issued in lower Austria forbidding manufacturers and tradesmen to sell nickel-plated cooking vessels. It is stated that vinegar and other acid substances dissolve nickel, and that this, in portions of one-seventh of a grain, causes vomiting, and is even more poisonous than copper.—*Sanitarian.*

